# Admission ECG changes predict short term-mortality after acute myocardial infarction less reliable in patients with diabetes

**DOI:** 10.1038/s41598-021-85674-9

**Published:** 2021-03-18

**Authors:** Timo Schmitz, Christian Thilo, Jakob Linseisen, Margit Heier, Annette Peters, Bernhard Kuch, Christa Meisinger

**Affiliations:** 1 MONIKA/KORA Myocardial Infarction Registry, University Hospital of Augsburg, Augsburg, Germany; 2 Chair of Epidemiology, LMU München at UNIKA-T Augsburg, Augsburg, Germany; 3 Department of Cardiology, University Hospital of Augsburg, Augsburg, Germany; 4 IRG Clinical Epidemiology, Helmholtz Zentrum München, Neuherberg, Germany; 5 KORA Study Centre, University Hospital of Augsburg, Augsburg, Germany; 6 Institute of Epidemiology, Helmholtz Zentrum München, Neuherberg, Germany; 7 German Center for Diabetes Research (DZD), Neuherberg, Germany; 8 Department of Internal Medicine, Hospital Nördlingen, Nördlingen, Germany

**Keywords:** Myocardial infarction, Cardiovascular biology, Cardiovascular diseases

## Abstract

Prior studies examined association between short-term mortality and certain changes in the admission ECG in acute myocardial infarction (AMI). Nevertheless, little is known about possible differences between patients with diabetes and without diabetes in this regard. So the aim of the study was to investigate the association between 28-day case fatality according to certain ECG changes comparing AMI cases with and without diabetes from the general population. From 2000 until 2017 a total of 9756 AMI cases was prospectively recorded in the study Area of Augsburg, Germany. Each case was assigned to one of the following groups according to admission ECG: ‘ST-elevation’, ‘ST-depression’, ‘only T-negativity’, ‘predominantly bundle branch block’, ‘unspecific changes’ and ‘normal ECG’ (the last two were put together for regression analyses). Multivariable adjusted logistic regression models were calculated to compare 28-day case-fatality between the ECG groups for the total sample and separately for diabetes and non-diabetes cases. For the non-diabetes group, the parsimonious logistic regression model revealed significantly better 28-day-outcome for the ‘normal ECG / unspecific changes’ group (OR: 0.47 [0.29–0.76]) compared to the reference group (STEMI). Contrary, in AMI cases with diabetes the category ‘normal ECG / unspecific changes’ was not significantly associated with lower short-term mortality (OR: 0.87 [0.49–1.54]). Neither of the other ECG groups was significantly associated with 28-day-mortality in the parsimonious logistic regression models. Consequently, the absence of AMI-typical changes in the admission ECG predicts favorable short-term mortality only in non-diabetic cases, but not so in patients with diabetes.

## Introduction

According to the presented admission ECG there are two main categories of acute myocardial infarction (AMI): ST-Elevation myocardial infarction (STEMI) and non-ST-myocardial infarction (NSTEMI). ST-elevations are the most typical changes in ECG in AMI. They go along with higher peak creatine kinase-MB (CK-MB) levels^1,2^, which is correlated with greater infarct size^3–8^. Nevertheless, several studies suggest that there is no greater difference between the two types in short-term mortality^1,9,10^, on the other hand some found more favorable prognosis for either STEMI or NSTEMI^11–14^.

The group of NSTEMIs can be further specified in several subgroups according to ECG; some researchers examined differences in mortality rates within the NSTEMI group^15–20^. Though, results from population-based registries are rare.

Type 2 diabetes is one of the major risk factors for cardiovascular diseases and AMI in particular^21^. Individuals with diabetes have a higher risk of heart attack than people without diabetes. Patients with diabetes, who have a heart attack, have a higher short- and long-term lethality than patients without diabetes^21^. Both groups also differ in terms of symptoms. Patients with diabetes seem to have more often atypical symptoms, less severe chest pain symptoms and more frequently suffer from a silent heart attack^22,23^. So the question arises, whether those two groups may also vary in the presented admission ECG and whether different ECG changes have different prospective value.

## Methods

### Data collection

The underlying data for this research was collected by the population-based KORA Myocardial Infarction Registry. It was established in 1984 as a part of the MONICA-project (Monitoring Trends and Determinants in Cardiovascular disease) and since 1995 it operates within the KORA (Cooperative Health Research in the region of Augsburg) framework as KORA Myocardial Infarction Registry. The study area consists of the city of Augsburg, Germany, and the two adjacent counties with a total of approximately 650,000 inhabitants.

For the Myocardial Infarction Registry Augsburg, potential cases of AMI are included when either diagnosis is confirmed by cardiac catheterization or patients have typical chest pain symptoms (angina pectoris) in combination with raised troponin levels and no other circumstances are more likely to explain the symptoms and medical findings. Doubtful cases are evaluated by trained physicians.

All cases of AMI that meet the above mentioned criteria are recorded, on condition the patient survived longer than 24 h, is 25–74 years old (from 2000 until 2008) or 25–84 years old (from 2009 until 2017) and has its primary residence within the study area. Consequently, the registration of all AMI cases within the study area minimizes the risk of severe bias. Trained study nurses carry out personal interviews using standardized questionnaire during the hospital stay. Further data collection is done by elaborating the patient’s medical files. In this way wide-ranging data for each case of AMI is collected including sociodemographic characteristics, risk factors, comorbidities, diagnostics and treatment.

Data collection of the MONICA/KORA MI registry has been approved by the ethics committee of the Bavarian Medical Association (Bayerische Landesärztekammer) and the study was performed in accordance with the Declaration of Helsinki. All study participants have given written informed consent.

For the analysis all consecutive cases between January 2000 until December 2017 were considered. Only patients with a first-time myocardial infarction were included. Cases with missing information on relevant covariates were excluded (except for left-ventricular ejection fraction (EF) and estimated glomerular filtration rate (eGFR)).

Admission ECG was evaluated by clinical physicians. Each case with available admission ECG was allocated to one of six groups according to the following principle: If there were significant ST-elevations present, the case was assigned to the STEMI group regardless of any other ECG changes. Significant ST-elevations were defined as new ST-segment elevations at the J point in 2 or more contiguous leads greater than 0.1 mV.

If significant ST-depressions were found in the remaining cases, they were classified as ‘ST-depression’ group, regardless of further ECG changes. The now remaining cases with T-negativity in 2 or more contiguous leads were assigned to the ‘T-negativity’ group. The leftover cases then were either assigned to the ‘normal ECG’ group (without any relevant ECG changes) or the ‘unspecific changes’ group including non-significant ST-segment changes, non-significant T-negativity or comparable changes.

The bundle branch block group consisted of all cases with right or left bundle branch with missing changes as mentioned above or bundle branch blocks with such great extent, that made it impossible to properly asses ST-segment and T wave changes.

Patients were assigned to the diabetes group, when either they reported diabetes as a pre-existing condition in the interview or diabetes was mentioned in the medical file. For this, it was not distinguished between different types of diabetes mellitus.

eGFR was calculated by admission creatinine levels according to the CKD-EPI formula. Four categories were defined: normal renal function (eGFR > 60 ml/min/1.73m^2^), slightly impaired renal function (eGFR between 30 and 60 ml/min/1.73m^2^), heavily impaired renal function (eGFR < 30 ml/min/1.73m^2^) and no information on renal function (values for creatinine levels were only available since 2005).

For the left-ventricular ejection fraction three categories were formed. Restricted left-ventricular ejection fraction (≤ 30%), normal left-ventricular ejection fraction (> 30%) and no-information on left-ventricular ejection fraction.

For the present analysis only patients with an incident myocardial infarction were included because earlier myocardial infarction can cause persisting ECG changes and so it might be difficult to differentiate between old changes due to prior events and changes caused by the current infarction.

The outcome of this study was 28-day case fatality after AMI. It was evaluated by checking the vital status of all registered persons on a regular basis. Therefore, death certificates were obtained from the local health departments.

### Statistical analysis

Baseline characteristics and potential covariates were cross-tabulated with the ECG groups. This was done for the total sample and for the diabetes and non-diabetes group separately. Categorical variables are presented as total number and percentages, continuous variables are described as median and interquartile range. To determine differences, Chi^2^ test for categorical variables and ANOVA (analysis of variance) for continuous variables were performed.

Logistic regression models were carried out to examine the associations between admission ECG and 28-day case-fatality. Due to the low number of events (death within 28 days after AMI) in the ‘normal ECG’ group and therefore relatively high statistical uncertainty of the results, the groups ‘normal ECG’ and ‘unspecific changes’ were combined together. Both groups can be considered as presentation of ECG without AMI-typical changes and consequently fit together pretty well.

First, a logistic regression model adjusted for sex and age was calculated. Thereafter, a parsimonious model was fitted using backwards elimination in order to avoid overfitting. Herefore, the following potential covariates were initially considered: sex, age, typical chest pain symptoms, smoking, hyperlipidemia, left-ventricular EF < 30%, impaired renal function, peak CK-MB levels, percutaneous coronary intervention (PCI), bypass and thrombolytic therapy. Bypass and thrombolytic therapy did not make a significant contribution to the model and consequently were removed as covariates. After all, the final models included only variables contributing significantly to the model (*p *value < 0.05). An exception from this was the variable sex, which did not contribute significantly but was forced to stay in the model.

All logistic regression models were performed for the entire cases and for the diabetes and non-diabetes group separately. Multicollinearity was checked by calculation of the variance inflation factor (VIF) for each covariate. VIF values did not exceed 2.5 for every covariate. There were about 2000 cases (from a total of 11,000) with missing information on peak-CK-MB-levels. Since just ignoring those cases could lead to a relevant bias, inverse probability weighting was implemented in the regression models, which were performed without those cases with missing information on peak-CK-MB-levels.

In addition to the regression models described above, all logistic regression models were also calculated with separated groups ‘normal ECG’ and ‘unspecific changes’. Results are displayed in the supplementary material.

### Ethics approval and consent to participate

Data collection of the MONICA/KORA MI registry has been approved by the ethics committee of the Bavarian Medical Association (Bayerische Landesärztekammer) and the study was performed in accordance with the Declaration of Helsinki. All study participants have given written informed consent.

## Results

Altogether 9756 patients with incident AMI were included in the analyses. 2115 cases with incidental AMI were excluded due to missing information on relevant covariates. Of all the patients included, 2934 had a physician-diagnosed self-reported diabetes (30.1%) and 6822 (69.9%) did not have any form of diabetes mellitus.

The biggest ECG-group was the STEMI group with 39.2% of all cases, followed by unspecific changes, normal ECG, ST-depression and bundle branch block (see Table 1). Diabetic patients have less frequently ST-elevation myocardial infarctions (35,0%) than individuals without diabetes (41,0%).

**Table 1:** Distribution of AMI cases by ECG group and diabetes diagnosis.

	Total number	ST-elevation	ST-depression	T-negativity	Unspecific changes	Normal ECG	Bundle branch block
Number of Total cases	9756	3825 (39.2%)	1085 (11.1%)	1222 (12.5%)	1687 (17.3%)	1235 (12.7%)	702 (7.2%)
Number of cases with diabetes	2934	1028 (35.0%)	401 (13.7%)	374 (12.7%)	537 (18.3%)	326 (11.1%)	268 (9.1%)
Number of non-diabetes cases	6822	2797 (41.0%)	684 (10.0%)	848 (12.4%)	1150 (16.8%)	909 (13.3%)	434 (6.4%)

Data presented as total number and row percentage.

Baseline characteristics of all subgroups are presented in Table 2. Significant differences between subgroups were seen for sex, age, hypertension, smoking, typical chest pain symptoms, left-ventricular ejection fraction, in-hospital complications, eGFR, peak CKMB levels, admission blood glucose, PCI, bypass and thrombolytic therapy. Hyperlipidemia only differed significantly for the non-diabetic patients within the ECG groups, but not for the patients with diabetes. Significant differences for admission troponin I were observed only among the diabetic AMI patients.

**Table 2:** Baseline characteristics of AMI cases by ECG group and diabetes diagnosis.

	ST–elevation	ST–depression	Only T–negativity	Unspecific changes	Normal ECG	Bundle branch block	*P* value	n
Female (n, %)	989 (25.9)	349 (32.2)	416 (34)	429 (25.4)	299 (24.2)	166 (23.6)	< 0.0001	9756
~ Diabetes	279 (27.1)	148 (36.9)	136 (36.4)	163 (30.4)	90 (27.6)	75 (28)	0.0007	2934
~ No diabetes	710 (25.4)	201 (29.4)	280 (33)	266 (23.1)	209 (23)	91 (21)	< 0.0001	6822
Age (years)*	61.2 (11.5)	66.2 (10.5)	64 (10.8)	64.4 (11)	61.5 (10.9)	69.2 (10)	< 0.0001	9756
~ Diabetes	63.7 (10.7)	68.7 (9.6)	66.1 (10.2)	67.2 (9.6)	64.2 (10)	70.6 (8.7)	< 0.0001	2934
~ No diabetes	60.3 (11.6)	64.7 (10.7)	63.1 (11)	63.1 (11.3)	60.5 (11)	68.3 (10.6)	< 0.0001	6822
Hypertension	2724 (71.2)	905 (83.4)	975 (79.8)	1340 (79.4)	937 (75.9)	594 (84.6)	< 0.005	9756
~ Diabetes	874 (85)	364 (90.8)	342 (91.4)	487 (90.7)	293 (89.9)	247 (92.2)	0.0002	2934
~ No diabetes	1850 (66.1)	541 (79.1)	633 (74.6)	853 (74.2)	644 (70.8)	347 (80)	< 0.005	6822
Hyperlipidemia	2235 (58.4)	687 (63.3)	740 (60.6)	1005 (59.6)	817 (66.2)	423 (60.3)	< 0.005	9756
~Diabetes	674 (65.6)	267 (66.6)	247 (66)	351 (65.4)	237 (72.7)	179 (66.8)	0.2714	2934
~ No diabetes	1561 (55.8)	420 (61.4)	493 (58.1)	654 (56.9)	580 (63.8)	244 (56.2)	0.0004	6822
Current smoker	1633 (42.7)	325 (30)	421 (34.5)	554 (32.8)	420 (34)	163 (23.2)	< 0.0001	9756
~ Diabetes	354 (34.4)	89 (22.2)	110 (29.4)	147 (27.4)	74 (22.7)	47 (17.5)	< 0.0001	2934
~ No diabetes	1279 (45.7)	236 (34.5)	311 (36.7)	407 (35.4)	346 (38.1)	116 (26.7)	< 0.0001	6822
Ex-smoker	1029 (26.9)	382 (35.2)	383 (31.3)	581 (34.4)	411 (33.3)	282 (40.2)	–	9756
~ Diabetes	323 (31.4)	156 (38.9)	123 (32.9)	208 (38.7)	138 (42.3)	119 (44.4)	–	2934
~ No diabetes	706 (25.2)	226 (33)	260 (30.7)	373 (32.4)	273 (30)	163 (37.6)	–	6822
Never smoker	1163 (30.4)	378 (34.8)	418 (34.2)	552 (32.7)	404 (32.7)	257 (36.6)	–	9756
~ Diabetes	351 (34.1)	156 (38.9)	141 (37.7)	182 (33.9)	114 (35)	102 (38.1)	–	2934
~ No diabetes	812 (29)	222 (32.5)	277 (32.7)	370 (32.2)	290 (31.9)	155 (35.7)	–	6822
Typical chest pain symptoms	3386 (88.5)	817 (75.3)	986 (80.7)	1317 (78.1)	1077 (87.2)	508 (72.4)	< 0.0001	9756
~ Diabetes	889 (86.5)	272 (67.8)	282 (75.4)	384 (71.5)	286 (87.7)	179 (66.8)	< 0.0001	2934
~ No diabetes	2497 (89.3)	545 (79.7)	704 (83)	933 (81.1)	791 (87)	329 (75.8)	< 0.0001	6822
Lef-ventricular EF < 30%	208 (5.4)	61 (5.6)	40 (3.3)	85 (5.0)	7 (0.6)	80 (11.4)	< 0.0001	9756
~ Diabetes	75 (7.3)	29 (7.2)	19 (5.1)	28 (5.2)	1 (0.3)	39 (14.6)	< 0.0001	2934
~ No diabetes	133 (4.8)	32 (4.7)	21 (2.5)	57 (5)	6 (0.7)	41 (9.4)	< 0.0001	6822
Left ventricular EF > 30%	2981 (77.9)	803 (74)	932 (76.3)	1240 (73.5)	984 (79.7)	467 (66.5)	–	9756
~ Diabetes	781 (76)	288 (71.8)	279 (74.6)	385 (71.7)	255 (78.2)	163 (60.8)	–	2934
~ No diabetes	2200 (78.7)	515 (75.3)	653 (77)	855 (74.3)	729 (80.2)	304 (70)	–	6822
No information on Left ventricular EF	636 (16.6)	221 (20.4)	250 (20.5)	362 (21.5)	244 (19.8)	155 (22.1)	–	9756
~ Diabetes	172 (16.7)	84 (20.9)	76 (20.3)	124 (23.1)	70 (21.5)	66 (24.6)	–	2934
~ No diabetes	464 (16.6)	137 (20)	174 (20.5)	238 (20.7)	174 (19.1)	89 (20.5)	–	6822
Any in-hospital complication	863 (22.6)	181 (16.7)	135 (11)	216 (12.8)	107 (8.7)	144 (20.5)	< 0.0001	9756
~ Diabetes	225 (21.9)	83 (20.7)	49 (13.1)	79 (14.7)	29 (8.9)	59 (22)	< 0.0001	2934
~ No diabetes	638 (22.8)	98 (14.3)	86 (10.1)	137 (11.9)	78 (8.6)	85 (19.6)	< 0.0001	6822
eGFR > 60 (ml/min/1.73m^2^)	2255 (59)	493 (45.4)	642 (52.5)	983 (58.3)	806 (65.3)	335 (47.7)	< 0.0001	9756
~ Diabetes	523 (50.9)	151 (37.7)	165 (44.1)	256 (47.7)	199 (61)	104 (38.8)	< 0.0001	2934
~ No diabetes	1732 (61.9)	342 (50)	477 (56.2)	727 (63.2)	607 (66.8)	231 (53.2)	< 0.0001	6822
eGFR 30-60 (ml/min/1.73m^2^)	564 (14.7)	273 (25.2)	206 (16.9)	376 (22.3)	165 (13.4)	198 (28.2)	–	9756
~ Diabetes	198 (19.3)	133 (33.2)	89 (23.8)	149 (27.7)	57 (17.5)	86 (32.1)	–	2934
~ No diabetes	366 (13.1)	140 (20.5)	117 (13.8)	227 (19.7)	108 (11.9)	112 (25.8)	–	6822
eGFR < 30 (ml/min/1.73m^2^)	73 (1.9)	57 (5.3)	53 (4.3)	85 (5)	15 (1.2)	57 (8.1)	–	9756
~ Diabetes	33 (3.2)	32 (8)	36 (9.6)	53 (9.9)	6 (1.8)	32 (11.9)	–	2934
~ No diabetes	40 (1.4)	25 (3.7)	17 (2)	32 (2.8)	9 (1)	25 (5.8)	–	6822
Missing information on eGFR	933 (24.4)	262 (24.1)	321 (26.3)	243 (14.4)	249 (20.2)	112 (16)	–	9756
~ Diabetes	274 (26.7)	85 (21.2)	84 (22.5)	79 (14.7)	64 (19.6)	46 (17.2)	–	2934
~ No diabetes	659 (23.6)	177 (25.9)	237 (27.9)	164 (14.3)	185 (20.4)	66 (15.2)	–	6822
Peak CK–MB (U/L)	118 (50–238)	45 (22–97)	31 (15–62)	40 (21–82)	34 (18–61)	42 (22–99)	< 0.0001	9756
~ Diabetes	103 (44–201)	37 (19–76)	28 (15–55)	38 (20–79)	34 (18–60)	35 (19–70)	< 0.0001	2934
~ No diabetes	123(53–249)	50 (25–111)	33 (16–66)	40 (21–82)	33 (18–62)	51 (23–120)	< 0.0001	6822
Admission Troponin I (ng/ml)	0.75(0.1–6.5)	0.68(0.14–3.5)	0.87(0.19–4.5)	0.47(0.12–2.4)	0.30(0.08–1.3)	0.75(0.16–3.7)	0.57	6016
~ Diabetes	0.93 (0.1–8.5)	0.71(0.16–3.8)	1.1 (0.21–5.1)	0.45(0.12–2.4)	0.25(0.09–0.9)	0.78(0.26–4.0)	< 0.0001	1818
~ No diabetes	0.69 (0.1–5.8)	0.67(0.13–3.5)	0.8(0.18–4.22)	0.51(0.12–2.6)	0.31(0.08–1.5)	0.63(0.13–3.4)	0.69	4198
Admission blood glucose(mg/dl)	144 (134–153)	138 (122–150)	142 (129–153)	143 (130–153)	145 (135–154)	140 (126–151)	< 0.0001	7684
~ Diabetes	143 (133–153)	135 (115–146)	140 (123–153)	138 (124–150)	144 (131–154)	135 (121–147)	< 0.0001	2322
~ No diabetes	144 (134–153)	141 (126–151)	142 (131–152)	145 (133–154)	146 (136–154)	142 (129–154)	< 0.0001	5362
PCI	3229 (84.4)	600 (55.3)	829 (67.8)	1069 (63.4)	900 (72.9)	423 (60.3)	< 0.0001	9756
~ Diabetes	846 (82.3)	204 (50.9)	247 (66)	317 (59)	233 (71.5)	146 (54.5)	< 0.0001	2934
~ No diabetes	2383 (85.2)	396 (57.9)	582 (68.6)	752 (65.4)	667 (73.4)	277 (63.8)	< 0.0001	6822
Bypass	341 (8.9)	274 (25.3)	213 (17.4)	287 (17.0)	152 (12.3)	108 (15.4)	< 0.0001	9756
~ Diabetes	100 (9.7)	94 (23.4)	62 (16.6)	97 (18.1)	40 (12.3)	43 (16.0)	< 0.0001	2934
~ No diabetes	241 (8.6)	180(26.3)	151(17.8)	190(16.5)	112(87.7)	65 (15.0)	< 0.0001	6822
Thrombolytic therapy	344 (9.0)	16 (1.5)	16 (1.3)	19 (1.1)	19 (1.5)	10 (1.4)	< 0.0001	9756
~ Diabetes	83 (8.1)	4 (1.0)	3 (0.8)	2 (0.4)	4 (1.2)	6 (2.2)	< 0.0001	2934
~ No diabetes	261 (9.3)	12 (1.8)	13 (1.5)	17 (1.5)	15 (1.7)	4 (0.9)	< 0.0001	6822

Categorical data presented as total numbers and % (proportion within each ECG group). Numeric data presented as median and (IQR). Chi2 test was used to calculate p-values for categorical data and ANOVA (analysis of variance) was used to calculated p-values for numeric data.

*Presented as mean (SD).

Categorical data presented as total numbers and % (proportion within each ECG group). Numeric data presented as median and (IQR). Chi2 test was used to calculate *p* values for categorical data and ANOVA (analysis of variance) was used to calculated *p* values for numeric data.

**Figure 1:**
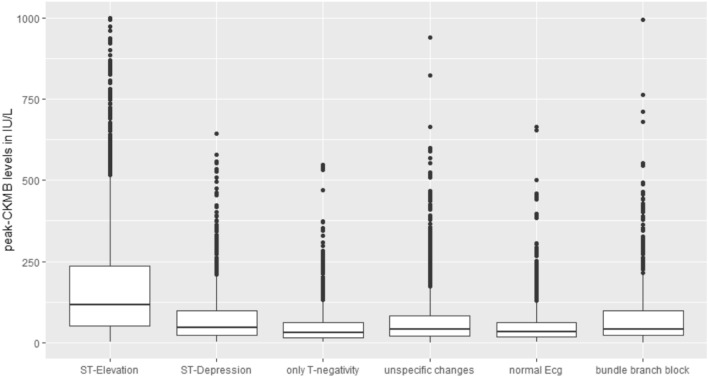
Peak CK-MB-levels in AMI cases by ECG group (in IU/L). Data represented by boxplots with median and 25% and 75% quartile. ST-Elevation has the highest median peak-CK-MB-level.

Figure 1 shows peak-CKMB-levels for the different ECG groups. The STEMI groups had noticeably higher peak-CKMB-levels compared to all other groups. The subgroups of the NSTEMI’s have comparable levels among each other (see Table 2).

**Figure 2:**
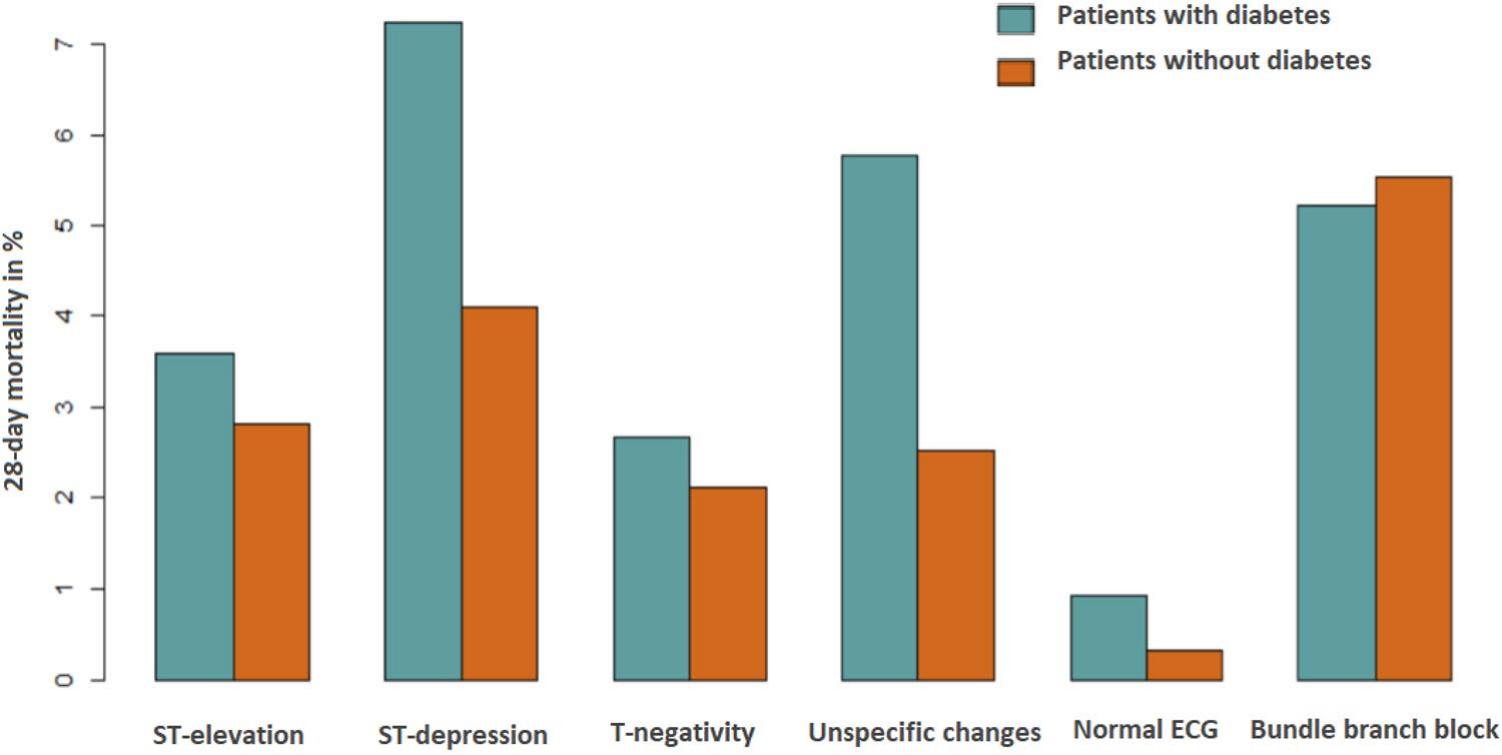
28-day fatality rates of AMI cases by ECG group and diabetes diagnosis (in %). 28-day-case fatality rates are higher for patients with diabetes for every ECG group except for the bundle branch block with a slightly higher 28-day-case fatality rate for patients without diabetes.

Table 3 displays the case fatality rates grouped by ECG. Overall, the highest mortality was seen in the bundle branch group and ST-depression group with 5.3% of fatal cases within 28-days. The normal ECG group had the lowest rate with 0.5%. Fatality rates for diabetic AMI patients were higher than the rates for the non-diabetes patients in every ECG group except for the bundle branch group (see Fig. 2).

**Table 3:** 28-day case fatality of AMI cases by ECG group and diabetes diagnosis.

	ST-elevation	ST-depression	T-negativity	Unspecific changes	Normal ECG	Bundle branch block
All cases	116 (3.0%)	57 (5.3%)	28 (2.3%)	60 (3.6%)	6 (0.5%)	38 (5.4%)
Diabetes	37 (3.6%)	29 (7.2%)	10 (2.7%)	31 (5.8%)	3 (0.9%)	14 (5.2%)
Non-diabetes	79 (2.8%)	28 (4.1%)	18 (2.1%)	29 (2.5%)	3 (0.3%)	24 (5.5%)

Data presented in total numbers and % of all cases.

For the total sample (diabetes and non-diabetes), the logistic regression model adjusted for sex and age revealed significantly higher short-term mortality for ST-depression (OR: 1.46 [1.07–2.00]) and significantly lower mortality for the normal ECG / unspecific changes group (OR: 0.71 [0.54–0.95]) compared to the STEMI group. The T-negativity and the bundle branch block group did not vary significantly from the reference group (see Table 4). The same logistic regression model was calculated with separate groups for normal ECG and unspecific changes, which is displayed in the supplementary material, Table 1. In this model, the normal ECG was associated with significantly lower short-term mortality than the STEMI group (0.18 [0.09–0.36]), but not so the unspecific changes group (OR: 1.08 [0.80–1.45]).

**Table 4:** Results of the logistic regression model for 28-day mortality of AMI cases by ECG group and diabetes diagnosis, adjusted for sex and age.

ECG	All cases		Diabetes		Non-diabetes	
	OR [95% CI]	p-value	OR [95% CI]	p-value	OR [95% CI]	p- value
STEMI	1		1		1	
ST-depression	1.46 [1.07–2.00]	0.017	1.75 [1.08–2.84]	0.023	1.23 [0.81–1.88]	0.334
T-negativity	0.75 [0.52–1.09]	0.131	0.79 [0.43–1.46]	0.459	0.73 [0.46–1.16]	0.185
Normal ECG/Unspecific changes	0.71 [0.54–0.95]	0.019	1.07 [0.69–1.67]	0.768	0.52 [0.35–0.76]	< 0.001
Bundle branch block	1.30 [0.91–1.87]	0.151	1.09 [0.60–2.00]	0.775	1.46 [0.93–2.29]	0.098

*OR* odds ratio, *95% CI* 95% confidence interval.

The parsimonious model was adjusted for sex, age, typical chest pain symptoms, smoking, hyperlipidemia, left-ventricular EF < 30%, impaired renal function (according to eGFR), peak CK-MB levels, admission glucose levels and PCI (see Table 5). In this model, calculated for all cases (diabetes and non-diabetes), only the ‘normal ECG / unspecific changes’ group had a significantly lower short-term-mortality (OR: 0.71 [0.54–0.95]). All other ECG groups did not differ significantly from the STEMI group.

**Table 5:** Results of the parsimonious logistic regression models* for 28-day mortality of AMI cases by ECG group and diabetes diagnosis.

ECG	All cases		Diabetes		Non-diabetes	
	OR [95% CI]	p-value	OR [95% CI]	p-value	OR [95% CI]	p-value
STEMI	1		1		1	
ST-depression	1.04 [0.72–1.5]	0.845	1.35 [0.75–2.41]	0.315	0.83 [0.50–1.38]	0.479
T-negativity	0.81 [0.52–1.24]	0.329	0.78 [0.38–1.61]	0.500	0.85 [0.50–1.46]	0.561
Normal ECG/Unspecific changes	0.6 [0.42–0.86]	0.005	0.87 [0.49–1.54]	0.641	0.47 [0.29–0.76]	0.0019
Bundle branch block	0.89 [0.59–1.35]	0.594	0.89 [0.45–1.78]	0.748	0.85 [0.49–1.46]	0.559

*OR* odds ratio, *95% CI* 95% confidence interval.

*Adjusted for sex, age, typical chest pain symptoms, smoking, hyperlipidemia, left-ventricular EF < 30%, impaired renal function (according to GFR), peak CK-MB levels, admission glucose levels, PCI.

The same regression models then were performed separately for diabetic and non-diabetic AMI patients. In the model including the AMI cases without diabetes, the normal ECG/ unspecific changes group had a significantly better short-term survival than the STEMI group (OR: 0.47 [0.29–0.76]). In the model including only cases with diabetes on the other hand, the ‘normal ECG / unspecific changes’ group did not differ significantly from the reference group (OR: 0.87 [0.49–1.54]). ST-Depression, T-negativity and bundle branch block were not significantly associated with 28-day case fatality in either of the parsimonious regression models.

Similar results can be obtained calculating the parsimonious models with separated groups for normal ECG and unspecific changes. Albeit based on fewer 28-day fatality cases in each group both ECG groups are significantly associated with lower short-term mortality compared to the STEMI group for the non-diabetic sample, but not so for the diabetes group (see supplementary material, Table 2).

## Discussion

### Short-term-mortality according to admission ECG (diabetes and non-diabetes)

Compared to numbers found in prior studies from the KORA Myocardial Infarction Registry, the 28-day fatality rates in this recent study are lower^24^. This applies in particular to the group of BBB. Kuch et. al found decreasing fatality rates for AMI from 1985 to 2004. The most striking decline was seen for the BBB group comparing the years 1995–1999 (25.3% 28-day case fatality) and 2000–2004 (10.3% case-fatality rate). In this study we found a further dropping case fatality rate of 5.4%. In 2002, troponin I began to be used routinely at the biggest hospital in the study region, which means considerable progresses in diagnostics. In consequence the proportion of non-ST-elevation AMI in the study population increased. Presumably also enhanced therapy over the last decades accounts for dropping 28-day case fatality rates. This includes more frequent and improved recanalization therapy (PCI and coronary artery bypass graft) and superior medical therapy^24^. It must be mentioned, that further differences may be due to different inclusion criteria, as we only admitted cases with first-time AMI into the analysis.

Regarding the crude 28-day-fatality the STEMI group was located in the midfield of all 6 ECG groups, with the ST-depression, unspecific changes and BBB associated with higher and the T-negativity and normal ECG group with lower 28-day case-fatality. ST-elevation was associated with higher peak-CK-MB levels than the other ECG groups. Higher CK-MB levels in STEMI were found in prior investigations as well^1,2^ and are thought to be representative for greater myocardial damage caused by hypoxia^3–8^. Nevertheless, ST-segment elevation is a class I indication for a primary PCI strategy^25^ and consequently in the STEMI group (84.4%) the frequency of PCI was higher than in any other ECG group (range from 55.3% to 72.9%). In-hospital cardiac catheterization is known to be associated with lower mortality, especially in high risk patients^26–28^. In addition, patients in the STEMI group were slightly younger at the event than the ones in the other groups. Faster and easier diagnostics, more frequent PCI and younger age might partly explain similar short-term outcomes in STEMI despite higher myocardial damage.

In a previous study from the KORA Myocardial Infarction Registry similar results were found in this regard. After adjusting for several important covariates, ST-Elevation was no longer a significant predictor for worse short-term outcome compared to the reference group (no or unspecific changes in ECG)^29^. Other studies came to similar conclusions as well^1,9,10^. Nevertheless, A. Marceau et. al performed a systematic review including 23 studies comparing short-term mortality for STEMI vs. NSTEMI in 2013, which revealed worse short-term prognosis for STEMI compared to NSTEMI^12^.

For the normal ECG, the unadjusted 28-case fatality rate was the lowest of all ECG groups. This trend remained in the model adjusted for sex and age and the parsimonious model (see supplementary material Table 1 and 2). Prior studies came to comparable results^30,31^.

BBB on the other hand was not independently associated with short-term-mortality in the parsimonious model. This differs from prior findings of this registry, where BBB was an independent predictor for adverse short-term outcomes compared to the reference group (no or unspecific changes) even after full adjustment^29^. But also in comparison to ST-elevation, mortality for BBB was found to be much higher. As discussed above, reasons for improved outcome in BBB might be advanced diagnostics (Troponin-I was established in 2002 in the largest hospital in the study area) and improved treatment. Other investigations and reviews also found worse short-term survival for patients with either right BBB, left BBB or both^32–35^. It has to be mentioned, that in the present study we could not distinguish between new onset BBB and preexisting BBB. Also we did not differentiate between right and left bundle branch block. Both aspects might attenuate predictive value for BBB in our analysis.

ST-depression was associated with a significantly higher 28-day-mortality in the model adjusted for sex and age. Nevertheless, this effect was no longer significant in the parsimonious model. Several studies have found that (specific) presentations of ST-depression had a negative predictive value for short-term-mortality^17,19,36^. Others didn’t find significant difference between ST-depression and ST-elevation concerning short-term mortality^37^, including the earlier study from the KORA Myocardial Infarction Registry^29^. Only-T-negativity didn’t reach significance in either of the models, which is comparable to the findings of our prior study, where no significant difference was seen between the groups of unspecific changes (including T-negativity) and ST-elevation^29^.

### Differences between diabetes and non-diabetes patients

The case distribution shows that patients with diabetes had slightly less often classical ST-elevation in the admission ECG (35% of all cases) compared to the patient without diabetes (41% of all cases). Nevertheless, the distribution of the cases according to the ECG groups was comparable between the diabetic and the non-diabetic patients.

As expected, we found that short-term mortality was higher for patients with diabetes than for patients without diabetes, which was also detected in many studies before^38–40^. A prior study from our registry found non-significantly higher 28-day case fatality in diabetic men and women compared to non-diabetic patients as well^41^.

For patients with diabetes, no ECG group was a significant predictor for short-term mortality after adjustment. In contrast, for non-diabetic patients, the ’normal ECG / unspecific changes’ group was significantly associated with lower short-term mortality in the parsimonious model, but not so in patients with diabetes. ST-depression, T-negativity and BBB were not significantly associated with short-term mortality in either group (diabetes or non-diabetes). To our knowledge, such differences regarding short-term-mortality according to ECG-groups stratified by diabetes were not investigated yet and so can’t be compared to previous results.

It can be concluded, that the absence of AMI-typical ECG changes like ST-elevation, ST-depression or T-negativity predicts favorable short-term outcomes less reliable for persons with diabetes compared to persons without diabetes. A possible explanation for this circumstance might be diverse characteristics of coronary artery disease (CAD) in patients with diabetes. First, the prevalence of multivessel-diseases could be higher among patients with diabetes and atherosclerosis tends to be more diffuse and severe^42–44^. Due to this differences, diabetic patients with severe CAD might be more likely to present unspecific ECG changes or no ECG changes at all. Furthermore, this particularities in CAD may complicate surgical or percutaneous intervention and in this way lead to incomplete revascularization or increase the risk of complication^45^. These aspects could at least partially explain a relatively high short-term mortality despite the absence of characteristic ECG changes. Comorbidities and risk factors associated with diabetes like platelet and coagulation abnormalities^45^, which we haven’t considered in the adjusted logistic regression models, may further contribute to increased mortality risks in diabetic patients with missing specific changes in the admission ECG.

Prospectively, improvements in this regard may come from new antidiabetic drugs like glucagon-like peptide-1 receptor agonists (GLP-1 RAs) and sodium-glucose co-transporter-2 (SGLT-2) inhibitors. It has been shown, that the use of those new medications has cardiovascular protective effects and can reduce cardiovascular events and deaths^46,47^. Perhaps, the increasing use of those drugs will enhance short-term outcomes for diabetic patients in AMI and especially for patients with unspecific ECG changes or no changes at all.

### Strengths and limitations

This study is characterized by some strengths. First to mention is the relatively high number of cases from a population-based registry with consecutive enrollment, which reduces the risk of selection bias. In addition to information on the actual event a large number of sociodemographic data, risk factors, comorbidities and information on in-hospital complications and treatment was collected. The fine subdivision in admission ECG assessment was performed by physicians and allowed further sub-classification in the NSTEMI group. Nevertheless, there are some limitations to our study. Although the selection of AMI cases was done very carefully, few cases might have been included which were not true myocardial infractions. To minimize their number, all doubtful cases were assessed by trained physicians. Since only patients up to 74 years (2000 until 2008) and up to 85 years (2009 until 2017) were included, results can’t be applied to older patients. Furthermore, the results may not be generalized to all ethnic groups since no information on ethnicity was available. Information on preexisting bundle branch block was not available as well. Moreover, we might not have considered all relevant confounders and cannot exclude possible reverse causation.

## Conclusion

Normal admission ECG is associated with lower short-term mortality in patients with incident AMI. The absence of AMI-typical changes in the admission ECG is an independent predictor of lower short-term mortality only for people without diabetes, but not for patients with diabetes. This should be considered in physician’s decision on acute therapy of the AMI.

## Supplementary Information

Supplementary Information

## Data Availability

The data will not be shared. Due to restrictions from Helmholtz Zentrum München, data are available upon request for any researcher based on a standard agreement on data provision within the KORA Research Platform.

## Abbreviations

ANOVA: Analysis of variance
AMI: Acute myocardial infarction
BBB: Bundle branch block
CAD: Coronary artery disease
CK-MB levels: Peak creatine kinase-MB levels
ECG: Electrocardiogram
EF: Left-ventricular ejection fraction
eGFR: Estimated glomerular filtration rate
MONICA-project: Monitoring trends and determinants in cardiovascular disease
NSTEMI: Non-ST-elevation myocardial infarction
PCI: Percutaneous coronary intervention
STEMI: ST-elevation myocardial infarction
VIF: Variance inflation factor
