# Supplementary material for: Admission ECG changes predict short term-mortality after acute myocardial infarction less reliable in patients with diabetes
Source: Sci Rep. 2021 Mar 18;11:6307. doi: 10.1038/s41598-021-85674-9 (PMC7973741; doi:10.1038/s41598-021-85674-9)
Supplement: Supplementary file 1 — Supplementary Information [file 41598_2021_85674_MOESM1_ESM.docx]

***Admission ECG changes predict short term-mortality after acute myocardial infarction less reliable in patients with diabetes.***

Timo Schmitz^1,2^, Christian Thilo^3^, Jakob Linseisen^2,4^, Margit Heier^5,6^, Annette Peters^6,7^, Bernhard Kuch^8*^ Christa Meisinger^2,4*^

^1^ MONIKA/KORA Myocardial Infarction Registry, University Hospital of Augsburg; ^2^Chair of Epidemiology, LMU München at UNIKA-T Augsburg; ^3^University Hospital of Augsburg, Department of Cardiology; ^4^ IRG Clinical Epidemiology, Helmholtz Zentrum München; ^5^University Hospital of Augsburg, KORA Study Centre, ^6^Institute of Epidemiology, Helmholtz Zentrum München; ^7^German Center for Diabetes Research (DZD) Neuherberg Germany, ^8^Department of Internal Medicine, Hospital Nördlingen, Germany

*shared last authorship

Correspondence to: Timo Schmitz, t.schmitz@unika-t.de

***Supplementary material***

***Table 1: Results of the logistic regression model for 28-day mortality of AMI cases by ECG group and diabetes diagnosis, adjusted for sex and age (separate groups for ‘normal ECG’ and ‘unspecific changes’).***

| ECG | All Cases  OR [95% CI] p-value | | Diabetes  OR [95% CI] p-value | | Non-Diabetes  OR [95% CI] p-value | |
| --- | --- | --- | --- | --- | --- | --- |
| STEMI | 1 |  | 1 |  | 1 |  |
| ST-depression | 1.48  [1.08-2.02] | 0.014 | 1,77  [1.10-2.87] | 0.02 | 1.24  [0.81-1.89] | 0.321 |
| T-negativity | 0.76  [0.52-1.09] | 0.138 | 0.8  [0.43-1.47] | 0.473 | 0.73  [0.46-1.17] | 0.19 |
| Unspecific changes | 1.08  [0.80-1.45] | 0.615 | 1.52  [0.96-2.40] | 0.074 | 0.81  [0.55-1.22] | 0.315 |
| Normal ECG | 0.18  [0.09-0.36] | < 0.001 | 0.31  [0.12-0.81] | 0.017 | 0.12  [0.04-0.33] | < 0.001 |
| Bundle branch block | 1.32  [0.92-1.89] | 0.13 | 1.11  [0.61-2.03] | 0.738 | 1.48  [0.94-2.31] | 0.088 |

OR: odds ratio, 95% CI: 95% confidence interval

***Table 2: Results of the parsimonious logistic regression models* for 28-day mortality of AMI cases by ECG group and diabetes diagnosis (separate groups for ‘normal ECG’ and ‘unspecific changes’).***

| ECG | All Cases  OR [95% CI] p-value | | Diabetes  OR [95% CI] p-value | | Non-Diabetes  OR [95% CI] p-value | |
| --- | --- | --- | --- | --- | --- | --- |
| STEMI | 1 |  | 1 |  | 1 |  |
| ST-depression | 1.05  [0.73-1.52] | 0.796 | 1.35  [0.76-2.41] | 0.309 | 0.84  [0.51-1.4] | 0.509 |
| T-negativity | 0.81  [0.53-1.25] | 0.344 | 0.78  [0.38-1.61] | 0.496 | 0.86  [0.5-1.47] | 0.581 |
| Unspecific changes | 0.74  [0.51-1.07] | 0.107 | 1.02  [0.57-1.83] | 0.956 | 0.59  [0.36-0.97] | 0.039 |
| Normal ECG | 0.26  [0.12-0.58] | 0.001 | 0.38  [0.11-1.3] | 0.123 | 0.21  [0.07-0.61] | 0.004 |
| Bundle branch block | 0.91  [0.6-1.38] | 0.657 | 0.9  [0.45-1.8] | 0.773 | 0.87  [0.5-1.49] | 0.602 |

OR: odds ratio, 95% CI: 95% confidence interval

* adjusted for sex, age, typical chest pain symptoms, smoking, hyperlipidemia, left-ventricular EF < 30%, impaired renal function (according to GFR), peak CK-MB levels, admission glucose levels, PCI
